# Adult attachment and acceptance-rejection in Colombia and Spain: maternal and paternal contributions across contexts

**DOI:** 10.3389/fpsyg.2026.1887282

**Published:** 2026-08-06

**Authors:** Silvia Perzolli, Adele Bernardi, Andrea Bizzego, Vincenzo Paolo Senese, Rodrigo Carcamo, Ana Angel-Echeverry, Giuseppe Iandolo, Gianluca Esposito

**Affiliations:** 1 Department of Psychology and Cognitive Science, University of Trento, Trento, Italy; 2 University of Campania Vanvitelli, Caserta, Italy; 3 Facultad de Psicología y Humanidades, Universidad San Sebastián, Sede Valdivia, Valdivia, Chile; 4 Doctorado en Ciencias Humanas, Universidad de Talca, Talca, Chile; 5 Universidad Europea de Madrid, Madrid, Spain

**Keywords:** adult attachment, caregiving, cross-cultural, parental acceptance-rejection, Colombia, Spain

## Abstract

**Objective:**

This study examined adult attachment and perceived parental acceptance-rejection in Colombia and Spain, focusing on country differences and on whether country moderated the associations between perceived maternal and paternal rejection and adult attachment dimensions.

**Method:**

Participants were 283 adults born in Colombia (*n* = 143) or Spain (*n* = 140). They completed the Experiences in Close Relationships-Revised scale and the Parental Acceptance-Rejection Questionnaire. Measurement invariance was supported across countries.

**Results:**

Colombian participants reported higher attachment anxiety [*t*(276.46) = 6.15, *p* < 0.001, *d* = 0.73], direct avoidance [*t*(279.28) = 3.31, *p* = 0.001, *d* = 0.39], maternal rejection [*t*(255.47) = 7.40, *p* < 0.001, *d* = 0.88], and paternal rejection [*t*(280.74) = 5.45, *p* < 0.001, *d* = 0.65] than Spanish participants. Perceived parental rejection was associated with greater attachment insecurity, although the pattern differed across attachment dimensions with paternal rejection showing the most consistent associations. Country moderated the association between paternal rejection and attachment anxiety and direct avoidance. Slope contrasts indicated that these associations were stronger among Colombian than Spanish participants for attachment anxiety (Δ*β* = 0.345, *p* = 0.005, adjusted *p* = 0.010) and direct avoidance (Δ*β* = 0.368, *p* = 0.006, adjusted *p* = 0.012).

**Conclusion:**

These findings suggest that the links between perceived parental rejection and adult attachment may vary across cultural contexts and highlight the importance of considering paternal relationships in cross-cultural attachment research.

## 1 Introduction

Attachment theory provides a central framework for understanding how early relational experiences contribute to individual differences in affect regulation, interpersonal expectations, caregiving, and close relationships across the life span (Ainsworth et al., 1978; Bowlby, 1969, 1982; Mikulincer and Shaver, 2007). According to Bowlby’s theory, repeated interactions with caregivers are progressively internalized as working models of the self and others, shaping expectations about whether significant others are available, responsive, and emotionally reliable (Bowlby, 1973; Bretherton, 1990; Mikulincer and Shaver, 2005, 2012). In adulthood, these representations are commonly assessed through self-report measures that examine how individuals experience, interpret and regulate closeness in current romantic relationships. The Experiences in Close Relationships scale - Revised scale (ECR-R), operationalizes adult attachment along two relatively independent dimensions: *attachment anxiety* and *attachment avoidance* (Fraley et al., 2011). Attachment anxiety reflects concerns about rejection, abandonment, and the availability of the partner, whereas attachment avoidance refers to discomfort with intimacy, dependence, and emotional vulnerability. Although the ECR-R assesses adult attachment as it is expressed in romantic relationships, rather than directly measuring childhood experiences with parents, attachment theory assumes a degree of continuity across relational systems (Opie et al., 2021; Pinquart et al., 2013). Adult attachment dimensions may therefore be meaningfully examined in relation to remembered caregiving experiences, particularly perceived parental acceptance-rejection, which captures how individuals recall the emotional quality of early care. Specifically, it examines whether caregivers are remembered as warm, affectionate, supportive, and accepting, or as hostile, neglectful, emotionally distant, or rejecting (Rohner, 2004; Rohner and Khaleque, 2005; Rohner and Lansford, 2017). Perceived parental rejection may include overt hostility or aggression, but also indifference, neglect, or a more diffuse sense of being unloved or unwanted, even in the absence of explicit maltreatment (Rohner, 2004; Rohner and Khaleque, 2005). In this sense, perceived parental acceptance-rejection can be understood as one relational pathway through which internal working models of the self and others may develop.

A further issue concerns whether maternal and paternal acceptance-rejection should be considered jointly or separately. Attachment and parenting research has historically focused primarily on mothers, partly reflecting the assumption that mothers are typically the main caregivers in early life (Bornstein, 2019; Mermelshtine and Barnes, 2016). However, increasing evidence suggests that fathers may contribute uniquely to children’s cognitive, social, and affective development (Fitzgerald et al., 2020). Maternal and paternal relationships may differ not only in caregiving involvement, but also in their relational meaning. The developmental significance of parental involvement is not reducible to the amount of time spent with the child; rather, the quality and nature of parent–child interactions appear central to adjustment (Amato and Rezac, 1994; Sarkadi et al., 2008). For this reason, examining maternal and paternal acceptance-rejection separately may clarify whether both parental relationships are similarly associated with adult attachment insecurity or whether they show distinct patterns of association.

These questions are particularly relevant in cross-cultural research. Attachment theory proposes that the need for protection and emotional security is a universal feature of human development, but the organization and expression of attachment are shaped by culturally embedded expectations about closeness, autonomy, dependence, emotional expression, parental authority, and family roles (Keller, 2013; Mesman et al., 2016). Cultural contexts may influence not only caregiving practices, but also the meanings individuals assign to parental warmth, rejection, emotional distance, and authority. Adult attachment patterns have been linked to country of origin, collectivism, ethnicity, and cultural identification, suggesting that attachment distributions are shaped by multiple and intersecting cultural processes rather than by national membership alone (Agishtein and Brumbaugh, 2013). Nevertheless, attachment research remains disproportionately based on North American and Western European samples, while Latin American contexts continue to be underrepresented despite increasing research activity in the region (Causadias and Posada, 2013). The comparison between Colombia and Spain offers a meaningful context for examining these issues. Both countries are Spanish-speaking, which reduces linguistic distance in the administration of Spanish-language measures, but they are not culturally interchangeable. Colombia represents a Latin American context in which family relationships may be strongly embedded in interdependence, intergenerational expectations, respect for adults, and family obligations. Spain, by contrast, is a Southern European context in which family relationships, parental involvement, and intergenerational ties are also culturally salient, but are embedded in different historical, social, and institutional conditions. Comparing these two country-of-birth groups therefore allows us to examine whether associations between perceived maternal and paternal acceptance-rejection and adult attachment dimensions are shared across Spanish-speaking groups or whether they vary according to cultural context. Despite the theoretical compatibility between attachment theory and parental acceptance-rejection theory, limited evidence is available on whether perceived maternal and paternal rejection are similarly associated with adult attachment dimensions across different Spanish-speaking cultural contexts. In particular, it remains unclear whether maternal and paternal rejection show distinct associations with adult attachment anxiety and avoidance, and whether these associations are similar across Colombian and Spanish participants or moderated by country of birth. Addressing this gap may contribute to a more culturally situated understanding of attachment by clarifying whether remembered parental acceptance-rejection is linked to adult attachment insecurity in similar ways across Colombia and Spain.

The present study therefore examined adult attachment and perceived parental acceptance-rejection among adults born in Colombia and Spain. Specifically, the study investigated the following research questions:

1. Do Spanish and Colombian participants differ in adult attachment anxiety and avoidance?
2. Do Spanish and Colombian participants differ in perceived maternal and paternal acceptance-rejection?
3. Are perceived maternal and paternal acceptance-rejection associated with adult attachment anxiety and avoidance?
4. Do maternal and paternal acceptance-rejection show distinct associations with adult attachment dimensions?
5. Does country moderate the association between maternal and paternal acceptance-rejection and adult attachment dimensions?

Based on attachment theory and interpersonal acceptance-rejection theory, higher perceived parental rejection was expected to be associated with greater attachment anxiety and avoidance, whereas higher perceived parental acceptance was expected to be associated with lower attachment insecurity (Bowlby, 1973; Mikulincer and Shaver, 2007; Rohner, 2004; Rohner and Khaleque, 2005; Rohner and Lansford, 2017). Because maternal and paternal roles may have distinct relational and cultural meanings, maternal and paternal acceptance-rejection were expected to show partially distinct associations with adult attachment dimensions (Rohner and Veneziano, 2001; Rohner and Lansford, 2017). Finally, given that Colombia and Spain represent distinct Spanish-speaking country contexts in which family organization, socialization values, and meanings of closeness, authority, and caregiving may differ, the study examined whether country of birth moderated the associations between perceived maternal and paternal acceptance-rejection and adult attachment anxiety and avoidance (Bornstein, 2012; Causadias and Posada, 2013; Di Giunta et al., 2024; Keller, 2013; Rey-Guerra et al., 2026).

## 2 Methods

### 2.1 Participants

For the present cross-cultural study, participants were included if they were adults, Spanish-speaking, and born in either Colombia or Spain. Participants were excluded if they did not meet the inclusion criteria, did not provide the informed consent or provided incomplete responses to the study variables. Missing data were handled through listwise exclusion. Duplicate responses were screened by checking repeated submissions with overlapping demographic information. Invalid responses, including incomplete, inconsistent, or plausible entries, were removed before analysis. The final sample consisted of 283 Spanish-speaking adults between 18 and 68 years of age (M = 33.97, SD = 11.10). The present cross-cultural analysis was derived from a larger Spanish-speaking sample. Colombia and Spain were selected because they were two theoretically relevant country-of-birth groups with large and balanced subsamples. The final analytic sample size was determined by the number of eligible participants available in the broader dataset.

The sample consisted primarily of women (77.74%), followed by men (20.85%), non-binary participants (0.35%), and participants who preferred not to disclose their gender identity (1.06%). Concerning country of birth, the sample was divided between Colombian (*n* = 143, 50.53%) and Spanish participants (*n* = 140, 49.47%). Most participants resided in Spain (*n* = 136, 48.06%) or Colombia (*n* = 128, 45.23%) at the time of data collection, while a smaller number reported residence in other countries. In terms of marital status, approximately half of the participants identified as single (*n* = 141, 49.82%). Participants in a romantic relationship represented 26.50% of the sample (*n* = 75), whereas 15.55% were married (*n* = 44), 7.77% were separated or divorced (*n* = 22), and one participant was widowed (0.35%). Regarding educational attainment, the largest proportion of participants reported having completed technical or professional studies (*n* = 89, 31.45%). Undergraduate degrees were reported by 26.86% of the sample (*n* = 76), while 22.61% had completed master’s degrees (*n* = 64). Most participants were employed (*n* = 186, 65.72%), whereas smaller proportions identified as students (*n* = 75, 26.50%), unemployed (*n* = 17, 6.01%), or retired (*n* = 5, 1.77%; See Table 1 for demographics).

**Table 1:** Sample demographic characteristics.

Variable	Category	*N* (%)/statistic
Age	Mean (SD) [min–max]	33.97 (11.1) [18–68]
Gender	1 = Female	220 (77.74%)
	2 = Male	59 (20.85%)
	4 = Prefer not to say	3 (1.06%)
	3 = Non-binary	1 (0.35%)
Marital status	1 = Single	141 (49.82%)
	2 = In a relationship	75 (26.5%)
	3 = Married	44 (15.55%)
	4 = Separated/divorced	22 (7.77%)
	5 = Widowed	1 (0.35%)
Education	5 = Technical/professional degree	89 (31.45%)
	6 = Bachelor’s degree	76 (26.86%)
	7 = Master’s degree	64 (22.61%)
	4 = High school diploma	32 (11.31%)
	8 = Doctorate	11 (3.89%)
	3 = Secondary education	4 (1.41%)
	9 = Other	4 (1.41%)
	2 = Primary education	2 (0.71%)
	1 = No degree	1 (0.35%)
Occupation	4 = Employed	186 (65.72%)
	3 = Student	75 (26.5%)
	2 = Unemployed	17 (6.01%)
	5 = Retired	5 (1.77%)
Primary language	4 = European Spanish	138 (48.76%)
	1 = Latin American Spanish	100 (35.34%)
	3 = South American Spanish	41 (14.49%)
	2 = Central American Spanish	2 (0.71%)
	5 = Other	2 (0.71%)
Country of birth	Colombia	143 (50.53%)
	Spain	140 (49.47%)
Country of residence	Spain	136 (48.06%)
	Colombia	128 (45.23%)
	Ecuador	2 (0.71%)
	United States	2 (0.71%)
	Italy	2 (0.71%)
	Germany	1 (0.35%)
	Argentina	1 (0.35%)
	Canada	1 (0.35%)
	Chile	1 (0.35%)
	Costa Rica	1 (0.35%)
	France	1 (0.35%)
	England	1 (0.35%)
	Ireland	1 (0.35%)
	Madrid	1 (0.35%)
	Mexico	1 (0.35%)
	Dominican Republic	1 (0.35%)
	Segovia	1 (0.35%)
	Turkey	1 (0.35%)

### 2.2 Procedure

Participants were recruited through a non-probabilistic convenience sampling strategy using social media platforms, university networks, and personal contacts. Data collection was conducted entirely online using a self-report questionnaire battery administered in Spanish. Participation was voluntary and anonymous, and no financial compensation was provided. Before accessing the questionnaires, participants received information regarding the aims of the study, confidentiality procedures, and the voluntary nature of participation. After providing informed consent, participants completed a demographic questionnaire followed by measures assessing adult attachment and perceived parental acceptance-rejection. The present study was part of a broader research project on attachment and relational experiences among Spanish-speaking adults. For this reason, the full online survey required approximately 20–30 min to complete. For the present cross-cultural analysis, the sample was restricted to participants born in Colombia and Spain, as these two national groups represented theoretically relevant Spanish-speaking cultural contexts. Each participant provided informed consent prior to participation. The study protocol was approved by the Ethics Committee of the University of Trento (protocol number: 2024-034ESA).

### 2.3 Measures

The study’s instruments consisted of three measures: a brief sociodemographic questionnaire, the Experiences in Close Relationships-Revised (ECR-R; Brennan et al., 1998; Fraley et al., 2000), and the Parental Acceptance-Rejection Questionnaire (PARQ; Rohner, 2004).

#### 2.3.1 Sociodemographic questionnaire

Participants reported age, gender, country of birth, country of residence, language, educational level, occupational status, and marital/relationship status. For the regression analyses, relationship status was recoded into a binary variable distinguishing participants who were currently in a romantic relationship or married from those who were single, separated/divorced, or widowed.

#### 2.3.2 Parental acceptance-rejection

Perceived parental acceptance-rejection was assessed using the Adult PARQ mother and father versions, based on the Spanish version validated by Rohner and Carrasco (2014). Participants completed the questionnaire separately for their mother and father, reporting how they remembered each parent’s behavior during childhood. Each version includes 24 items assessing four dimensions: Warmth/Affection, Hostility/Aggressiveness, Indifference/Neglect, and Undifferentiated Rejection. Responses were provided on a 4-point Likert scale, from *1 = Almost never true* to *4 = Almost always true,* in accordance with the Spanish version validated by Rohner and Carrasco (2014). To calculate total parental rejection scores, the warmth/affection dimension was reverse-scored according to standard procedures. Therefore, higher total scores indicate a greater perception of parental rejection. Mother and father total scores were computed separately.

#### 2.3.3 Adult attachment

Adult attachment was assessed using the Experiences in Close Relationships-Revised (ECR-R; Brennan et al., 1998; Fraley et al., 2000), using the Colombian Spanish adaptation proposed by Zambrano et al. (2009). The ECR-R is a widely used self-report measure designed to assess attachment-related anxiety and attachment-related avoidance in close relationships. Participants were instructed to answer the items with reference to their experiences in romantic relationships. The ECR-R includes 36 items rated on a 5-point Likert scale ranging from *5 = Always* to *1 = Never*, consistent with the Colombian adaptation proposed by Zambrano et al. (2009). The ECR-R is theoretically based on two broad dimensions: attachment anxiety reflects fears of rejection, abandonment, and excessive need for reassurance, whereas attachment avoidance reflects discomfort with intimacy, emotional closeness, and dependency on others. In the present study, and consistent with psychometric work on Spanish-language ECR-R adaptations (Zambrano et al., 2009; Nóblega et al., 2018), we examined an empirical three-factor structure distinguishing attachment anxiety, direct avoidance, and reverse-worded avoidance items. The reverse-worded avoidance factor was not interpreted as a separate theoretical attachment dimension, but as an empirical measurement component reflecting the behavior of reverse-worded avoidance items in the present data.

### 2.4 Analytic plan

All analyses were conducted in RStudio using the *tidyverse, psych, lavaan, semTools, effsize, broom, car,* and *emmeans* packages. Preliminary analyses included demographic summaries, descriptive statistics, and internal consistency estimates for all relevant scales. Descriptive statistics included means, standard deviations, medians, ranges, skewness, and kurtosis. Reliability was evaluated using Cronbach’s alpha for the total sample and separately by country. Before testing cross-cultural differences, measurement invariance was examined across Colombian and Spanish participants for the ECR-R and PARQ. For the ECR-R, a three-factor empirical model was specified, corresponding to attachment anxiety, direct avoidance, and reverse avoidance. Although the ECR-R is theoretically based on two broad dimensions, anxiety and avoidance, the conventional two-factor model was also tested and showed substantially poorer fit in the present data. Therefore, and consistent with Spanish-language psychometric studies of the ECR-R, the three-factor empirical specification was retained for the main analyses. For the PARQ, separate four-factor models were estimated for maternal and paternal forms, including warmth, hostility, indifference, and undifferentiated rejection. Configural, metric, and scalar invariance models were estimated using confirmatory factor analysis in *lavaan*, with the WLSMV estimator, theta parameterization, and ordinal indicators. Model fit was evaluated using scaled χ^2^, CFI, TLI, RMSEA, and SRMR. Changes in CFI and RMSEA were inspected across increasingly constrained models, with invariance considered supported when ΔCFI was ≤0.010 and ΔRMSEA was ≤0.015. Absolute fit indices were also considered when interpreting the adequacy of the measurement models.

The normality of the study variables was assessed separately by country using the Shapiro–Wilk test. To test hypotheses 1 and 2, cross-cultural differences between Colombian and Spanish participants were then examined using Welch independent-samples *t*-tests for ECR-R attachment dimensions and PARQ maternal and paternal total and subscale scores. Cohen’s *d* was calculated as an effect size. Because several variables deviated from normality, Mann–Whitney U tests were also conducted as non-parametric sensitivity analyses. Bonferroni corrections were applied to adjust for multiple comparisons.

To test hypotheses 3 and 4, multiple linear regression models were estimated separately for attachment anxiety, direct avoidance, and reverse avoidance. Maternal and paternal PARQ total scores were entered simultaneously to evaluate their unique contributions. Continuous predictors and dependent variables were standardized as z-scores before regression analyses, so coefficients are reported in standardized units. Country of birth was coded with Colombia as the reference category. Relationship status was coded as partnered/married versus not partnered. For each attachment outcome, two final models were compared: (1) an additive model including maternal and paternal rejection, country of birth, age, gender, and relationship status (2) and a country moderation model including the interactions between maternal and paternal rejection and country of birth, with the same covariates. Model comparison was based on AIC, *R*^2^, and adjusted *R*^2^. To directly examine whether maternal and paternal acceptance-rejection showed distinct associations with adult attachment dimensions, maternal and paternal regression coefficients were compared within the simultaneous regression models using linear hypothesis tests. When interaction effects were examined, simple slopes were estimated using *emmeans*, and slope contrasts between Colombian and Spanish participants were tested. Bonferroni corrections were applied within each family of related tests. A sensitivity power analysis was conducted to estimate the minimum detectable effect size given the final sample size. With *N* = 283 and up to eight regression terms in the final models, the study had 80% power at *α* = 0.05 to detect an overall regression effect of approximately *f*^2^ = 0.055 and a single-term effect of approximately *f*^2^ = 0.029.

## 3 Results

Preliminary analyses through Cronbach’s α indicated good to excellent reliability across all study variables, both in the total sample and within each country group, supporting the adequacy of the measures used in the present sample (See Table 2). Measurement invariance analyses were conducted before testing cross-cultural differences. For the ECR-R and the maternal and paternal PARQ models, changes in model fit across configural, metric, and scalar models met the predefined criteria for invariance, with ΔCFI values within 0.010 and ΔRMSEA values within 0.015 (See Table 3). Specifically, configural invariance supported the same factorial structure across Colombian and Spanish participants; metric invariance indicated that factor loadings were comparable across groups; and scalar invariance indicated that item thresholds were sufficiently comparable across groups. However, some absolute fit indices, particularly RMSEA and SRMR for the ECR-R and paternal PARQ, were less than optimal. Thus, the invariance results supported cross-country comparability according to change-in-fit criteria, although cross-country comparisons should be interpreted with caution. Shapiro–Wilk tests indicated significant deviations from normality for several study variables within both country groups. Therefore, Welch independent-samples *t*-tests were used for group comparisons, and Mann–Whitney U tests were conducted as non-parametric sensitivity analyses.

**Table 2:** Cronbach’s alpha by country and country *t*-tests for ECR-R/PARQ variables.

Scale	Alpha	Alpha Col.	Alpha Spain	Colombia M (SD)	Spain M (SD)	*t*(df)	*p*	Bonf. *p*	*d*
PARQ Warmth Mother	0.93	0.93	0.89	17.43 (6.61)	12.29 (4.72)	*t*(257.07) = 7.54	<0.001***	<0.001***	0.89
PARQ Hostility Mother	0.90	0.90	0.88	12.97 (5.58)	9.45 (4.00)	*t*(257.55) = 6.10	<0.001***	<0.001***	0.72
PARQ Indifference Mother	0.87	0.84	0.85	13.94 (4.77)	10.43 (3.91)	*t*(272.64) = 6.77	<0.001***	<0.001***	0.80
PARQ Undifferentiated Rejection Mother	0.88	0.86	0.87	8.01 (3.70)	5.97 (2.73)	*t*(261.38) = 5.28	<0.001***	<0.001***	0.63
PARQ Total Mother	0.96	0.96	0.95	52.34 (18.70)	38.14 (13.18)	*t*(255.47) = 7.40	<0.001***	<0.001***	0.88
PARQ Warmth Father	0.94	0.93	0.94	19.80 (6.83)	15.11 (6.89)	*t*(280.75) = 5.75	<0.001***	<0.001***	0.68
PARQ Hostility Father	0.87	0.85	0.90	10.84 (4.79)	9.71 (4.26)	*t*(278.45) = 2.10	=0.036*	=0.472	0.25
PARQ Indifference Father	0.87	0.83	0.88	15.34 (4.89)	12.00 (4.73)	*t*(280.95) = 5.84	<0.001***	<0.001***	0.69
PARQ Undifferentiated Rejection Father	0.88	0.88	0.86	7.24 (3.63)	5.97 (2.77)	*t*(265.33) = 3.30	=0.001**	=0.014*	0.39
PARQ Total Father	0.95	0.94	0.95	53.21 (16.51)	42.79 (15.68)	*t*(280.74) = 5.45	<0.001***	<0.001***	0.65
ECR Anxiety	0.95	0.95	0.94	41.36 (14.16)	31.71 (12.18)	*t*(276.46) = 6.15	<0.001***	<0.001***	0.73
ECR Direct Avoidance	0.88	0.82	0.93	11.55 (4.76)	9.62 (5.05)	*t*(279.28) = 3.31	=0.001**	=0.014*	0.39
ECR Reverse Avoidance	0.91	0.85	0.94	19.36 (6.55)	17.61 (8.53)	*t*(260.76) = 1.93	=0.054	=0.708	0.23

Alpha = Cronbach’s alpha. Bonf. *p* = Bonferroni-corrected *p*-value. *p <0.05; **p < 0.01; ***p < 0.001.

**Table 3:** Measurement invariance by country.

Scale	Model	Chi-square	df	*p*	CFI	TLI	RMSEA	SRMR	Delta CFI	Delta RMSEA
ECR-R	Configural	1,571.48	642	<0.001	0.952	0.948	0.102	0.106	−0.001	−0.001
ECR-R	Metric	1,605.32	666	<0.001	0.952	0.949	0.100	0.114		
ECR-R	Scalar	1,648.71	720	<0.001	0.952	0.953	0.096	0.107	0.001	−0.004
PARQ Mother	Configural	1,035.11	492	<0.001	0.958	0.953	0.089	0.079		
PARQ Mother	Metric	1,006.07	512	<0.001	0.962	0.959	0.083	0.090	0.004	−0.006
PARQ Mother	Scalar	1,085.52	536	<0.001	0.958	0.956	0.085	0.079	−0.004	0.003
PARQ Father	Configural	1,290.33	492	<0.001	0.934	0.926	0.107	0.104		
PARQ Father	Metric	1,270.09	512	<0.001	0.938	0.933	0.103	0.111	0.003	−0.005
PARQ Father	Scalar	1,349.78	536	<0.001	0.933	0.931	0.104	0.104	−0.005	0.001

To test hypotheses 1 and 2, independent-samples *t*-tests showed significant differences between Colombian and Spanish participants in adult attachment dimensions. Colombian participants reported higher attachment anxiety than Spanish participants, *t*(276.46) = 6.15, *p* < 0.001, *d* = 0.73. They also reported higher direct attachment avoidance, *t*(279.28) = 3.31, *p* = 0.001, *d* = 0.39. Both effects remained significant after Bonferroni correction. The difference in reverse avoidance was not significant after correction, *t*(260.76) = 1.93, *p* = 0.054, Bonferroni-adjusted *p* = 0.708.

Significant country differences also emerged for perceived parental rejection. Colombian participants reported higher perceived maternal rejection than Spanish participants, *t*(255.47) = 7.40, *p* < 0.001, *d* = 0.88, and higher perceived paternal rejection, *t*(280.74) = 5.45, *p* < 0.001, *d* = 0.65 (See Table 2). Both PARQ total-score differences remained significant after Bonferroni correction. Overall, Hypotheses 1 and 2 were supported: Colombian participants reported higher adult attachment insecurity, especially anxiety and direct avoidance, as well as higher perceived maternal and paternal rejection.

To test hypothesis 3, regression models were estimated separately for attachment anxiety, direct avoidance, and reverse avoidance. Maternal and paternal rejection were entered simultaneously, together with country, age, gender, and relationship status as covariates. For each outcome, an additive model with covariates was compared with a country moderation model including the interactions between maternal and paternal rejection and country. Model comparison was based on AIC, R^2^, and adjusted R^2^.

For attachment anxiety, the country moderation model showed the best fit, AIC = 719.94, adjusted *R*^2^ = 0.283. In this model, maternal rejection was positively associated with attachment anxiety, *β* = 0.169, SE = 0.073, *t* = 2.326, *p* = 0.021, Bonferroni-adjusted *p* = 0.166. Paternal rejection was also positively associated with attachment anxiety, *β* = 0.346, SE = 0.078, *t* = 4.413, *p* < 0.001, Bonferroni-adjusted *p* < 0.001. Relationship status was negatively associated with attachment anxiety, *β* = −0.585, SE = 0.109, *t* = −5.371, *p* < 0.001, Bonferroni-adjusted *p* < 0.001. The interaction between paternal rejection and country was also significant, *β* = −0.345, SE = 0.121, *t* = −2.842, *p* = 0.005, Bonferroni-adjusted *p* = 0.039. For direct avoidance, the country moderation model also showed the best fit, AIC = 769.61, adjusted R^2^ = 0.145. Paternal rejection was positively associated with direct avoidance, *β* = 0.362, SE = 0.086, *t* = 4.223, *p* < 0.001, Bonferroni-adjusted *p* < 0.001. Age and relationship status were associated with direct avoidance at the uncorrected level, but neither association remained significant after Bonferroni correction: age, *β* = 0.156, SE = 0.061, *t* = 2.541, *p* = 0.012, Bonferroni-adjusted *p* = 0.093; relationship status, *β* = −0.299, SE = 0.119, *t* = −2.512, *p* = 0.013, Bonferroni-adjusted *p* = 0.101. The interaction between paternal rejection and country was significant, *β* = −0.368, SE = 0.132, *t* = −2.776, *p* = 0.006, Bonferroni-adjusted *p* = 0.047. For reverse avoidance, the additive model with covariates showed the best fit, AIC = 788.43, adjusted *R*^2^ = 0.080. Paternal rejection was positively associated with reverse avoidance, *β* = 0.180, SE = 0.067, *t* = 2.689, *p* = 0.008, Bonferroni-adjusted *p* = 0.046. Age showed a positive association, *β* = 0.128, SE = 0.063, *t* = 2.019, *p* = 0.044, but this association did not survive Bonferroni correction, Bonferroni-adjusted *p* = 0.267. Relationship status showed a negative association, *β* = −0.383, SE = 0.123, *t* = −3.112, *p* = 0.002, Bonferroni-adjusted *p* = 0.012. Overall, Hypothesis 3 was partially supported: perceived parental rejection appeared to be associated with adult attachment dimensions, although the pattern differed across outcomes. Paternal rejection showed the most consistent association across the final models.

To test hypothesis 4, regression coefficients for PARQ Mother and PARQ Father were directly compared within the simultaneous models. These comparisons did not provide evidence that paternal rejection was statistically stronger than maternal rejection for attachment anxiety, *F* = 0.004, *p* = 0.952, direct avoidance, *F* = 0.549, *p* = 0.459, or reverse avoidance, *F* = 1.272, *p* = 0.260. Hypothesis 4 received descriptive but not statistical support: paternal rejection showed a more consistent pattern of significant associations across outcomes, but formal coefficient comparisons did not indicate statistically significant differences between maternal and paternal effects.

To test hypothesis 5, moderation analyses were conducted to examine whether participants’ country of birth moderated the associations between parental rejection and adult attachment dimensions. Interaction terms between country and maternal and paternal acceptance-rejection scores were included in the regression models. No significant moderation effects emerged for maternal rejection. The interaction between maternal rejection and country was not significant for attachment anxiety (*β* = − 0.084, *p* = 0.532), direct avoidance (*β* = − 0.235, *p* = 0.111), or reverse avoidance (*β* = − 0.061, *p* = 0.688).

In contrast, the results provided evidence that country moderated the association between paternal rejection and anxiety (*β* = −0.345, *p* = 0.005, Bonferroni-adjusted *p* = 0.039) and direct attachment avoidance (*β* = −0.368, *p* = 0.006, Bonferroni-adjusted *p* = 0.047). No significant moderation effects emerged for reverse avoidance (*β* = 0.131, *p* = 0.342; See Table 4). Follow-up simple slopes analyses indicated that paternal rejection was more strongly associated with adult attachment insecurity among Colombian participants than among Spanish participants. Specifically, among Colombian participants, higher paternal rejection was significantly associated with higher attachment anxiety (*β* = 0.346, *SE* = 0.078, 95% *CI* [0.192, 0.501]) and higher direct avoidance (*β* = 0.362, *SE* = 0.086, 95% *CI* [0.193, 0.531]). In contrast, these associations were weak and non-significant among Spanish participants for both attachment anxiety (*β* = 0.002, *SE* = 0.093, 95% *CI* [−0.181, 0.184]) and direct avoidance (*β* = − 0.006, *SE* = 0.101, 95% *CI* [−0.205, 0.193]; See Table 5). Slope contrasts confirmed that the association between paternal rejection and anxiety was stronger in Colombia than in Spain, Δ*β* = 0.345, SE = 0.121, *t*(274) = 2.842, *p* = 0.005, Bonferroni-adjusted *p* = 0.010, and that the association between paternal rejection and direct avoidance was also stronger in Colombia than in Spain, *Δβ* = 0.368, *SE* = 0.132, *t*(274) = 2.776, *p* = 0.006, Bonferroni-adjusted *p* = 0.012. No significant country difference emerged for reverse avoidance, Δ*β* = 0.131, SE = 0.138, *t*(274) = 0.953, *p* = 0.342, Bonferroni-adjusted *p* = 0.683 (See Table 6). Taken together, Hypothesis 5 was partially supported: country significantly moderated the association between paternal rejection and attachment anxiety and direct avoidance, with this association being stronger among Colombian participants.

**Table 4:** AIC-selected models and significant associations.

Outcome	AIC-selected model	AIC	*R*^2^ adj.	Predictor	Beta	SE	*t*	*p*	Bonf. *p*
Anxiety	Country moderation	719.94	0.283	PARQ Mother	0.169	0.073	2.326	=0.021*	=0.166
Anxiety	Country moderation	719.94	0.283	PARQ Father	0.346	0.078	4.413	<0.001***	<0.001***
Anxiety	Country moderation	719.94	0.283	Relationship status (partnered/married vs. not partnered)	−0.585	0.109	−5.371	<0.001***	<0.001***
Anxiety	Country moderation	719.94	0.283	PARQ Father x Country (Spain vs. Colombia)	−0.345	0.121	−2.842	=0.005**	=0.039*
Direct avoidance	Country moderation	769.61	0.145	PARQ Father	0.362	0.086	4.223	<0.001***	<0.001***
Direct avoidance	Country moderation	769.61	0.145	Age	0.156	0.061	2.541	=0.012*	=0.093
Direct avoidance	Country moderation	769.61	0.145	Relationship status (partnered/married vs. not partnered)	−0.299	0.119	−2.512	=0.013*	=0.101
Direct avoidance	Country moderation	769.61	0.145	PARQ Father x Country (Spain vs. Colombia)	−0.368	0.132	−2.776	=0.006**	=0.047*
Reverse avoidance	Additive controls	788.43	0.080	PARQ Father	0.180	0.067	2.689	=0.008**	=0.046*
Reverse avoidance	Additive controls	788.43	0.080	Age	0.128	0.063	2.019	=0.044*	=0.267
Reverse avoidance	Additive controls	788.43	0.080	Relationship status (partnered/married vs. not partnered)	−0.383	0.123	−3.112	=0.002**	=0.012*

Regression terms with *p* < 0.05 are reported from the lowest-AIC model for each outcome; Bonferroni-corrected *p*-values are provided as an additional check. *p < 0.05; **p < 0.01; ***p < 0.001.

**Table 5:** Final simple slopes by country.

Outcome	Predictor	Country	Beta	SE	df	95% CI	Significant slope
Anxiety	PARQ Mother	Colombia	0.169	0.073	274	[0.026, 0.312]	Yes
Anxiety	PARQ Mother	Spain	0.253	0.114	274	[0.028, 0.479]	Yes
Direct avoidance	PARQ Mother	Colombia	0.065	0.079	274	[−0.092, 0.221]	No
Direct avoidance	PARQ Mother	Spain	0.300	0.125	274	[0.054, 0.546]	Yes
Reverse avoidance	PARQ Mother	Colombia	0.033	0.083	274	[−0.129, 0.196]	No
Reverse avoidance	PARQ Mother	Spain	0.095	0.130	274	[−0.161, 0.350]	No
Anxiety	PARQ Father	Colombia	0.346	0.078	274	[0.192, 0.501]	Yes
Anxiety	PARQ Father	Spain	0.002	0.093	274	[−0.181, 0.184]	No
Direct avoidance	PARQ Father	Colombia	0.362	0.086	274	[0.193, 0.531]	Yes
Direct avoidance	PARQ Father	Spain	−0.006	0.101	274	[−0.205, 0.193]	No
Reverse avoidance	PARQ Father	Colombia	0.236	0.089	274	[0.060, 0.411]	Yes
Reverse avoidance	PARQ Father	Spain	0.105	0.105	274	[−0.102, 0.312]	No

**Table 6:** Final slope contrasts.

Outcome	Predictor	Contrast	Estimate	SE	*t*	*p*	Bonf. *p*
Anxiety	PARQ Mother	Colombia - Spain	−0.084	0.135	−0.625	=0.532	=1.000
Direct avoidance	PARQ Mother	Colombia - Spain	−0.235	0.147	−1.601	=0.111	=0.221
Reverse avoidance	PARQ Mother	Colombia - Spain	−0.061	0.153	−0.401	=0.688	=1.000
Anxiety	PARQ Father	Colombia - Spain	0.345	0.121	2.842	=0.005**	=0.010*
Direct avoidance	PARQ Father	Colombia - Spain	0.368	0.132	2.776	=0.006**	=0.012*
Reverse avoidance	PARQ Father	Colombia - Spain	0.131	0.138	0.953	=0.342	=0.683

Contrasts compare the estimated slope in Colombia with the estimated slope in Spain. *p < 0.05; **p < 0.01; ***p < 0.001.

## 4 Discussion

The present study aimed to investigate adult attachment from a cross-cultural perspective by examining differences between Colombian and Spanish participants in attachment dimensions and perceived parental acceptance-rejection, as well as the associations between these experiences across adult attachment domains. Overall, the findings suggest that adult attachment is not only shaped by individual relational experiences but is also embedded within broader cultural and familial contexts that may influence how closeness, emotional dependence, and interpersonal distance are experienced and regulated (Keller, 2013, 2018; Mesman et al., 2016). Consistent with the first hypothesis, Colombian participants reported significantly higher levels of attachment anxiety and direct avoidance compared to Spanish participants. Importantly, the differences emerged specifically for attachment anxiety and direct avoidance, whereas reverse avoidance did not significantly differ after correction. This pattern may indicate that the cross-cultural differences observed in the present sample are more strongly related to explicit discomfort with closeness and fear of rejection rather than to the reverse avoidance dimension. However, because the present study used country of birth as a proxy for a cultural context, these interpretations should be considered contextual hypotheses rather than direct evidence of specific cultural mechanisms. Significant country differences also emerged for perceived maternal and paternal rejection, with Colombian participants reporting higher levels of both. These findings suggest that the Colombian group, at least within the present sample, perceived relational experiences characterized by higher levels of parental rejection. Within the PARQ framework, these scores should be interpreted specifically as perceived parental acceptance-rejection, including warmth, hostility, indifference/neglect, and undifferentiated rejection. Broader interpretations involving parental authority, emotional obligation, or psychological control are plausible in light of the cultural literature (Di Giunta et al., 2024; Mogro-Wilson and Cifuentes, 2021; Rey-Guerra et al., 2026), but they were not directly measured in this study and should therefore be treated cautiously.

The regression models further supported an association between perceived parental rejection and adult attachment dimensions. Paternal rejection showed the most consistent associations across attachment dimensions. However, formal coefficient comparisons did not demonstrate statistically significant differences between maternal and paternal effects; therefore, further evidence is needed to clarify whether maternal and paternal rejection may have distinct associations with adult attachment dimensions. This finding is noteworthy given the historical focus on maternal caregiving, despite growing evidence of father’s unique role in child’s development (Fitzgerald et al., 2020). Nevertheless, the absence of statistically significant differences between maternal and paternal coefficients suggests that both caregiving relationships may contribute to adult attachment insecurity in partially overlapping ways, consistent with multidimensional perspectives on attachment development emphasizing the combined influence of multiple attachment relationships (Howes and Spieker, 2008; Dagan and Sagi-Schwartz, 2018). Moderation analyses provided additional nuance to these findings. Specifically, country significantly moderated the association between paternal rejection and both attachment anxiety and direct avoidance, with paternal rejection showing a stronger association among Colombian participants and this association appeared weaker among Spanish participants. However, this moderation should be interpreted specifically as a country difference in the slope linking paternal rejection to attachment dimensions, rather than as evidence that paternal rejection was statistically stronger than maternal rejection overall. Therefore, the moderation findings should be interpreted specifically as country differences in the association between paternal rejection and attachment anxiety/direct avoidance, rather than as evidence that country moderates all links between parental rejection and adult attachment. One possible interpretation is that paternal rejection may acquire different relational meanings across cultural and family contexts. In some family-oriented cultural contexts, closeness, authority, emotional restraint, and role expectations may intersect in ways that shape how paternal rejection is experienced and linked to adult attachment insecurity. However, the present study did not directly measure family hierarchy, paternal authority, familism, interdependence, or emotional obligation. Therefore, these explanations remain interpretive hypotheses for future research rather than conclusions directly demonstrated by the data. Finally, the findings regarding paternal rejection are theoretically relevant, but the adjusted *R*^2^ values were modest. This indicates that perceived parental rejection accounts for only part of the variability in adult attachment dimensions, and that other individual, relational, and cultural factors not assessed in the present study are likely to contribute to attachment anxiety and avoidance.

### 4.1 Limitations

Several limitations should be acknowledged when interpreting the present findings. First, the sample was non-probabilistic, and recruited online through convenience sampling, which limits representativeness and generalizability In addition, no *a priori* power analysis was conducted for this specific country comparison. Although gender was included as a covariate in the final regression models, the sample was unbalanced with women representing the majority of participants. Given that maternal and paternal roles may be interpreted differently depending on participants’ gender and cultural background, future studies should recruit more gender-balanced samples and examine whether these associations vary across men, women and gender-diverse participants. The relatively high educational level of the sample should also be considered, as recruitment through social media, university networks, and personal contacts may have introduced educational, socioeconomic, and topic-interest biases. Second, the study used country of birth as a proxy for cultural context. Although Colombia and Spain represent two theoretically meaningful Spanish-speaking contexts, country of birth cannot capture individual-level cultural values, family norms, cultural identification, familism, independence-interdependence, or parental authority beliefs. Some participants also resided outside their country of birth at the time of data collection, which may have introduced migration or acculturation effects. In addition, both Colombia and Spain are internally heterogeneous contexts. Future studies should consider regional, socioeconomic, and cultural variation within each country, including possible differences between northern and southern Spain in the meaning of interpersonal closeness, dependence, and family relationships. Third, the study relied exclusively on retrospective self-report measures collected at a single time point. As a result, the findings should be interpreted as associations rather than causal evidence. Current attachment insecurity may influence how participants recall parental acceptance-rejection, and responses may also be affected by memory bias, current emotional functioning, negative affectivity, response style, or social desirability. Future research should use longitudinal designs, multiple informants, interviews, observational methods, and multi-method assessments to better clarify the direction and robustness of these associations. Fourth, relationship status may influence responses to the ECR-R, which assesses attachment in romantic relationships. We addressed this issue by including relationship status as a covariate in the final models, distinguishing participants who were partnered or married from those who were not partnered. However, the study did not assess relationship history, relationship duration, relationship quality, or current partner involvement in detail. Future studies should include more refined relational indicators. Fifth, the study did not collect sufficiently detailed information about family structure, parental absence, or the degree of maternal and paternal involvement. Future studies should assess family structure and parental involvement more directly, especially given the central role of parental rejection in the present findings. Finally, some measurement issues should be considered. The Colombian Spanish adaptation of the ECR-R was used for both Colombian and Spanish participants. Although both groups were Spanish-speaking and measurement invariance analyses were conducted, dialectal differences may have affected item interpretation. Future studies should compare country-specific Spanish adaptations and further examine the factorial structure of the ECR-R and PARQ across Spanish-speaking contexts.

Despite these limitations, the present study contributes to the growing literature examining attachment processes outside predominantly WEIRD (Western, Educated, Industrialized, Rich, and Democratic) populations (Henrich et al., 2010). The findings support the importance of considering attachment as a culturally situated relational process shaped by family dynamics, parental experiences, and broader sociocultural contexts. In particular, the results highlight the potential relevance of paternal relational experiences in shaping attachment strategies and suggest that the psychological impact of parent rejection may vary across cultural environments. Understanding how attachment processes are shaped across diverse relational and cultural systems may ultimately contribute to a more inclusive and context-sensitive attachment theory.

## 5 Conclusion

The present study contributes to a more culturally situated understanding of adult attachment by showing that the links between perceived parental rejection and attachment insecurity may vary across relational and cultural contexts. Rather than suggesting a uniform association between early caregiving experiences and adult attachment, the findings indicate that maternal and paternal rejection may hold partially distinct meanings, particularly when considered across different family systems. The stronger associations between paternal rejection and both attachment anxiety and direct avoidance among Colombian participants highlight the need to examine fathers not only as secondary caregivers, but as relational figures whose perceived rejection may be meaningfully associated with adult attachment insecurity. Future research should investigate how culturally embedded expectations about motherhood, fatherhood, closeness, and autonomy shape the pathways by which early family experiences carry over into adult intimate relationships.

## Data Availability

The raw data supporting the conclusions of this article will be made available by the authors, without undue reservation.

## References

[ref1] Agishtein P. Brumbaugh C. (2013). Cultural variation in adult attachment: the impact of ethnicity, collectivism, and country of origin. J. Soc. Evol. Cult. Psychol. 7, 384–405. doi: 10.1037/h0099181

[ref2] Ainsworth M. D. S. Blehar M. C. Waters E. Wall S. (1978). Patterns of Attachment: A Psychological Study of the Strange Situation. Hillsdale, NJ: Erlbaum.

[ref3] Amato P. R. Rezac S. J. (1994). Contact with nonresident parents, interparental conflict, and children’s behavior. J. Fam. Issues 15, 191–207. doi: 10.1177/0192513X94015002003

[ref4] Bornstein M. H. (2012). Cultural approaches to parenting. Parenting 12, 212–221. doi: 10.1080/15295192.2012.683359, 22962544 PMC3433059

[ref5] Bornstein M. H. (2019). “Parenting infants,” in Handbook of Parenting, ed. Bornstein M. H., vol. 1. Children and parenting. 3rd ed (Routledge), 3–55.

[ref6] Bowlby J. (1969). Attachment and Loss: Vol. 1. Attachment. New York: Basic Books.

[ref7] Bowlby J. (1973). Attachment and Loss: Vol. 2. Separation: Anxiety and Anger. New York: Basic Books.

[ref8] Bowlby J. (1982). Attachment and loss: retrospect and prospect. Am. J. Orthopsychiatry 52, 664–678. doi: 10.1111/j.1939-0025.1982.tb01456.x, 7148988

[ref10] Brennan K. A. Clark C. L. Shaver P. R. (1998). “Self-report measurement of adult attachment: an integrative overview,” in Attachment Theory and close Relationships, eds. Simpson J. A. Rholes W. S. (New York: Guilford Press), 46–76.

[ref11] Bretherton I. (1990). “Open communication and internal working models: their role in the development of attachment relationships,” in Nebraska Symposium on Motivation, ed. Thompson R. A., vol. 36 (Lincoln: University of Nebraska Press), 59–113. 3078938

[ref12] Causadias J. M. Posada G. (2013). The relevance of cross-national studies on early attachment: research advances in Latin America. Int. J. Behav. Dev. 63, 7–12.

[ref13] Dagan O. Sagi-Schwartz A. (2018). Early attachment network with mother and father: an unsettled issue. Child Dev. Perspect. 12, 115–121. doi: 10.1111/cdep.12272

[ref14] Di Giunta L. Uribe Tirado L. M. Ruiz Garcia M. (2024). Cultural values, parenting and child adjustment in Colombia. Int. J. Psychol. J. Int. Psychol. 59, 578–587. doi: 10.1002/ijop.13120, 38418410 PMC11257817

[ref15] Fitzgerald H. E. von Klitzing K. Cabrera N. J. Scarano de Mendonça J. Skjøthaug T. (2020). Handbook of Fathers and child Development: Prenatal to Preschool. Springer.

[ref16] Fraley R. C. Heffernan M. E. Vicary A. M. Brumbaugh C. C. (2011). The experiences in close relationships-relationship structures questionnaire: a method for assessing attachment orientations across relationships. Psychol. Assess. 23, 615–625. doi: 10.1037/a0022898, 21443364

[ref17] Fraley R. C. Waller N. G. Brennan K. A. (2000). An item response theory analysis of self-report measures of adult attachment. J. Pers. Soc. Psychol. 78, 350–365. doi: 10.1037//0022-3514.78.2.350, 10707340

[ref18] Henrich J. Heine S. J. Norenzayan A. (2010). The weirdest people in the world? Behav. Brain Sci. 33, 61–83. doi: 10.1017/S0140525X0999152X, 20550733

[ref19] Howes C. Spieker S. (2008). “Attachment relationships in the context of multiple caregivers,” in Handbook of Attachment: Theory, Research, and Clinical Applications, eds. Cassidy J. Shaver P. R. (New York: Guilford Press), 317–332.

[ref20] Keller H. (2013). Attachment and culture. J. Cross-Cult. Psychol. 44, 175–194. doi: 10.1177/0022022112472253

[ref21] Keller H. (2018). Universality claim of attachment theory: children’s socioemotional development across cultures. Proc. Natl. Acad. Sci. 115, 11414–11419. doi: 10.1073/pnas.1720325115, 30397121 PMC6233114

[ref23] Mermelshtine R. Barnes J. (2016). Maternal responsive-didactic caregiving in play interactions with 10-month-olds and cognitive development at 18 months. Infant Child Dev. 25, 296–316. doi: 10.1002/icd.1961

[ref24] Mesman J. van IJzendoorn M. H. Sagi-Schwartz A. (2016). “Cross-cultural patterns of attachment: universal and contextual dimensions,” in Handbook of Attachment: Theory, Research, and Clinical Applications, eds. Cassidy J. Shaver P. R.. 3rd ed (New York: Guilford Press), 852–877.

[ref25] Mikulincer M. Shaver P. R. (2005). Attachment security, compassion, and altruism. Curr. Dir. Psychol. Sci. 14, 34–38. doi: 10.1111/j.0963-7214.2005.00330.x

[ref26] Mikulincer M. Shaver P. R. (2007). Attachment in Adulthood: Structure, Dynamics, and change. New York: Guilford Press.

[ref27] Mikulincer M. Shaver P. R. (2012). An attachment perspective on psychopathology. World Psychiatry 11, 11–15. doi: 10.1016/j.wpsyc.2012.01.003, 22294997 PMC3266769

[ref29] Mogro-Wilson C. Cifuentes A. Jr. (2021). The influence of culture on Latino fathers’ parenting styles. J. Soc. Soc. Work Res. 12, 705–729. doi: 10.1086/715440, 35958683 PMC9360920

[ref30] Nóblega M. Núñez del Prado J. Alcántara N. Barreda V. A. Cabrerizo P. Castañeda E. A. . (2018). Propiedades psicométricas de una versión en español del Experiences in Close Relationships–Revised (ECR-R). Revista de Psicología 27, 1–13. doi: 10.5354/0719-0581.2018.52308

[ref31] Opie J. E. McIntosh J. E. Esler T. B. Duschinsky R. George C. Schore A. N. . (2021). Early childhood attachment stability and change: a meta-analysis. Attach Hum. Dev. 23, 897–930. doi: 10.1080/14616734.2020.1800769, 32772822 PMC7612040

[ref32] Pinquart M. Feußner C. Ahnert L. (2013). Meta-analytic evidence for stability in attachments from infancy to early adulthood. Attach Hum. Dev. 15, 189–218. doi: 10.1080/14616734.2013.746257, 23210665

[ref33] Rey-Guerra C. Borbón J. Guerra M. (2026). Gendered attitudes and parenting practices of mothers and fathers in two culturally diverse Colombian cities. Infant Child Dev. 35:e70068. doi: 10.1002/icd.70068

[ref34] Rohner R. P. (2004). The parental “acceptance-rejection syndrome”: universal correlates of perceived rejection. Am. Psychol. 59, 830–840. doi: 10.1037/0003-066X.59.8.830, 15554863

[ref35] Rohner R. Carrasco M. A. (2014). Teoría de la aceptación-rechazo interpersonal (IPARTheory): Bases conceptuales, método y evidencia empírica. Acción Psicológica 11, 9–26. doi: 10.5944/ap.11.2.14172

[ref36] Rohner R. P. Khaleque A. (2005). “Personality assessment questionnaire (PAQ): test manual,” in Handbook for the Study of parental Acceptance and Rejection, eds. Rohner R. P. Khaleque A.. 4th ed (Storrs, CT: Rohner Research Publications), 187–225.

[ref37] Rohner R. P. Lansford J. E. (2017). Deep structure of the human affectional system: introduction to interpersonal acceptance-rejection theory. J. Fam. Theory Rev. 9, 426–440.

[ref38] Rohner R. P. Veneziano R. A. (2001). The importance of father love: history and contemporary evidence. Rev. Gen. Psychol. 5, 382–405. doi: 10.1037/1089-2680.5.4.382

[ref39] Sarkadi A. Kristiansson R. Oberklaid F. Bremberg S. (2008). Fathers' involvement and children's developmental outcomes: a systematic review of longitudinal studies. Acta paediatrica (Oslo, Norway: 1992) 97, 153–158. doi: 10.1111/j.1651-2227.2007.00572.x, 18052995

[ref40] Zambrano R. Villada Zapata J. Vallejo V. J. Córdoba V. Giraldo J. J. Herrera B. . (2009). Propiedades psicométricas de la prueba de apego adulto “Experiencias en relaciones cercanas-revisado” (Experiences in Close Relationships–Revised, ECR-R) en población colombiana. Pensando Psicología 5, 6–14.

